# Spatial Variability of *Escherichia coli* in Rivers of Northern Coastal Ecuador

**DOI:** 10.3390/w7020818

**Published:** 2015-02-13

**Authors:** Gouthami Rao, Joseph N. S. Eisenberg, David G. Kleinbaum, William Cevallos, Gabriel Trueba, Karen Levy

**Affiliations:** 1 Department of Environmental Health, Emory University, 1518 Clifton Rd, Atlanta, GA 30322, USA; 2 Department of Epidemiology, University of Michigan, M5065 SPH II, 1415 Washington Heights, Ann Arbor, MI 48109-2029, USA; 3 Department of Epidemiology, Emory University, 1518 Clifton Rd, Atlanta, GA 30322, USA; 4 Centro de Biomedicina, Universidad Central del Ecuador, Carrera de Medicina Sodiro N14-121 e Iquique, Quito, Ecuador; 5 Universidad San Francisco de Quito, Diego de Robles y Vía Interoceánica, Quito 170157, Ecuador

**Keywords:** water quality, *Escherichia coli*, hydrodynamics, Ecuador

## Abstract

The use of contaminated surface water continues to be a pressing issue in areas of the world where people lack improved drinking water sources. In northern coastal Ecuador, many communities rely on untreated surface water as their primary source of drinking water. We undertook a study to explore how microscale river hydrodynamics affect microbial water quality at community water collection locations at three rivers with varying stream velocity and turbidity profiles. To examine how the distance from river shore and physiochemical water quality variables affect microbial contamination levels in the rivers; we collected a total of 355 water samples within six villages on three rivers; and tested for *Escherichia coli* concentrations using the IDEXX Quanti-tray method. We found that log_10_ *E. coli* concentrations decreased with increasing distance from shore (β = −0.017; *p* = 0.003). Water in the main channel had *E. coli* concentrations on average 0.12 log_10_ lower than within eddies along the river shore and 0.27 log_10_ lower between the sample closest to shore and any sample >6 m from the shore. Higher *E. coli* concentrations were also significantly associated with increased turbidity (β = 0.003; *p* < 0.0001) and decreased dissolved oxygen levels (β = −0.310; *p* < 0.0001). The results of this study can help inform community members about the safest locations to collect drinking water and also provide information on watershed scale transport of microbial contaminants between villages.

## 1. Introduction

Diarrhea is the fourth leading cause of mortality around the world, killing approximately 1.4–1.9 million people in 2010 [1,2]. The use of contaminated water continues to be a pressing issue in developing countries; approximately 1.8 billion people globally use a source of drinking water which suffers from fecal contamination [3] and 187 million people rely on untreated surface water [4]. Much of this water is consumed without adequate treatment.

Higher concentrations of microbial contaminants in surface water occur at locations near human populations, at observed at points of municipal sewage discharge [5], as well as at areas of activities such as bathing or washing [6]. Limited freshwater resources force many to use and reuse water sources, and in developing countries this contamination of water sources may lead to human exposure through washing/bathing or consumption of untreated water (e.g., [6–8]).

The effect of oxygenation and other physiochemical parameters on the die-off of fecal indicators has been extensively studied under laboratory environments; but there is limited research on how far contamination plumes reach within surface sources of drinking water, and what roles velocity, oxygenation, and other physiochemical characteristics of water have on die-off of microorganisms in natural environments. More work has been done on temporal [9–11] and spatial variability [11–13] in coastal environments than on streams [14], especially in tropical settings [15]. Understanding the impact of these factors on localized contamination processes in regions where people depend on untreated surface sources of drinking water may provide insights that can be used to improve water collection practices.

In this study, we explore these issues through a consideration of the spatial variability of microbial contamination on three different rivers of the Esmeraldas Province in northern coastal Ecuador. In this region, most human activities occur on the riverbanks, and contaminated water recirculates in eddies close to shore. The primary goal of this study was to understand how localized stream hydrodynamics, such as velocity of the channel, presence of eddies along the shore, and physiochemical parameters of the water, affect the microbiological quality of surface sources of drinking water. We tested the extent to which microbial contamination changes with increasing distance from shore (moving towards the central flow of a river), presence of turbulent eddies, and physiochemical parameters of water quality.

## 2. Materials and Methods

### 2.1. Study Region

This study was carried out in northern coastal Ecuador, in Esmeraldas Province, in association with a larger study on diarrheal disease transmission that has been ongoing since 2003 [16]. In this region, 125 villages line the banks of the Santiago, Onzole, and Cayapas Rivers. Household surveys that we previously conducted found that approximately 68% relied on untreated surface water as their primary source of drinking water [17], and on average 29% of households report treating their drinking water. Villagers also routinely access the river for bathing, washing and recreation, and only 46% of households report access to improved sanitation [18]. Through these human activities, the rivers become contaminated with fecal material.

Our investigation took place in six villages along three different rivers in the region. These three rivers represented a gradient of riverine conditions: the Santiago River has fast-flowing water, bedrock substrate, and clear waters; the Cayapas River has intermediate conditions; and the Onzole River has slow-flowing water, muddy substrate, and, is highly turbid. The structure and cultures of the human communities living along the three rivers are similar.

### 2.2. Study Design

**Figure 1:**
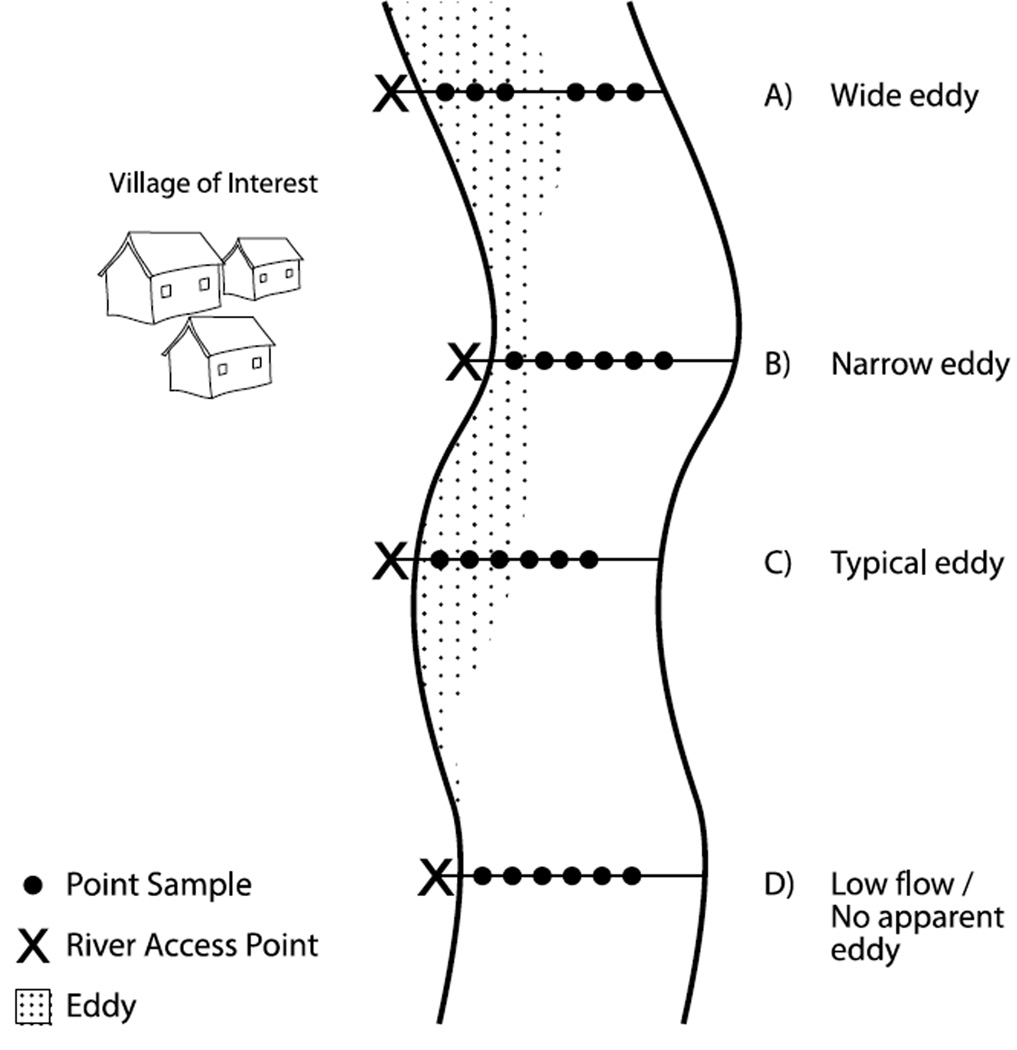
Field sampling schematic. At each village of interest, we sampled at sites along the banks of the river where the population accesses the river. We established river transects perpendicular to the river bank and took samples 2 m apart either within or outside of the eddy. Four different situations occurred, depending on the geometry of the river: (**A**) Wide eddy: when the eddy was >6 m wide, we took three samples within the eddy (each collected 2 m apart) and three samples outside of the eddy (each collected 2 m apart), with a gap between the third and fourth sample; (**B**) Narrow eddy: If the eddy was <6 m wide, we took as many samples as possible from within the eddy and the remainder outside of the eddy, with all samples collected 2 m apart; (**C**) Typical eddy: If the eddy was 6 m, we took three samples from within and three samples outside of the eddy, with all samples collected 2 m apart; (**D**) No apparent eddy: If there was no apparent eddy due to low flow, all samples were collected 2 m apart.

In each community, we identified two to three locations where villagers wash clothes and dishes, bathe, and collect water for drinking and household purposes. The majority of sites were located at the base of community stairways, which serve as river access points. At each river access site, we established a transect perpendicular to the shoreline, along which we collected six point samples, both within eddies (recirculating water near the shoreline) and outside of eddies, in the main stream channel (Figure 1). Eddies and eddy boundaries were determined by visual assessment. Samples were collected by boat, and distance from shore was measured using a Yardage Pro range-finder (Bushnell, Overland Park, KS, USA). Distances were validated by checking the range finder’s value three to four times before a point sample was collected.

In locations where there was an eddy along the shore at the sampling site, samples were taken 2 m apart within the eddy for the width of the eddy, and the remaining samples were taken 2 m apart beyond the eddy line (*n* = 156). If the eddy size was wider than 6 m, three samples were taken within the eddy, each 2 m apart, and three beyond the eddy, each line 2 m apart; in this case a gap existed between the last eddy sample and first sample outside of the eddy (*n* = 120). In locations with no flow (*i.e.*, no distinguishable eddy), all six samples were taken 2 m apart (*n* = 18). If the eddy size was less than 6 m, one or two samples were taken 2 m apart within the eddy, and the remaining outside the eddy (*n =* 48). The sampling design is shown in Figure 1.

### 2.3. Water Sample Characterization

Samples were collected during three field visits over 24 sampling days between 5 June and 19 July 2012. Water samples were collected between 10:00 and 11:00 am in Whirl-Pak bags (Nasco, Fort Atkinson, WI, USA), stored on ice, and tested for *Escherichia coli* within 6–8 h of collection using the IDEXX Quanti-tray most probable number (MPN) method (IDEXX, Westbrook, ME, USA). A negative control sample was also processed every day using sterilized water. Trays were incubated at 41 ± 3 °C for 18–24 h in a small portable incubator (Boekel, Feasterville, PA, USA). When centralized energy was not available, a generator was used to maintain power. On one day, voltage in the community was too low (<220 V) for the sealer to turn on, so a conventional iron was used to seal the trays, ensuring that all wells contained sample water (*n* = 18 samples). If turbidity levels were visibly high, we performed a 1:10 dilution by using syringes to extract 10 mL of the river water sample and adding to 90 mL of sterile water, in order to avoid plates with values too numerous to count (TNTC).

Physiochemical water quality measurements were also taken at the time of water sample collection. All probes were calibrated before each field visit. Temperature (°C) and pH were measured using a waterproof handheld device (Hannah Instruments, Woonsocket, RI, USA). Turbidity (Nephelometric Turbidity Units; NTU) was measured using a Hach 2100Q turbidimeter (Hach Company, Loveland, CO, USA). Dissolved Oxygen (DO_2_; ppm) was measured using a YSI handheld probe (YSI Inc., Yellow Springs, OH, USA). Instantaneous velocity was measured with a Flow Probe (Global Water Instrumentation Inc., Model FP111, College Station, TX, USA).

### 2.4. Statistical Analysis

Data analysis was conducted using SAS v9.3 (Cary, NC, USA), and graphics were produced in STATA v12 (College Station, TX, USA). A total of 332/355 (93.5%) samples fell within a countable *E. coli* range. Twelve (3.4%) samples were above the detection limit and treated as the maximum countable 2419.6 MPN/100 mL. Eleven samples (3.1%) were under the detection limit and treated as 0.5 MPN/100 mL, halfway between 0 and the lower detection limit of 1 MPN/100 mL. All *E. coli* concentrations were log_10_-transformed for analysis.

Simple linear regression and scatter plots were used to evaluate univariate relationships of water quality parameters (turbidity, stream velocity, temperature, and DO_2_) and distance from shore *versus* the continuous outcome variable of log_10_ *E. coli* concentration. Additionally, a correlated linear mixed modeling process was carried out, using an autoregressive (AR1) correlation structure, with transect defined as the cluster variable and included as a random intercept. This approach takes into account both autocorrelation by transect, and serial correlation by sampling day along each transect. Log_10_ concentration of *E. coli* was the primary outcome, distance from shore was the primary exposure variable, and temperature, DO_2_, turbidity, dichotomized velocity (>0 *vs.* 0 m/s), and the interactions of each of these variables with distance from shore were evaluated as potential confounders. Collinearity between the variables was assessed using a collinearity macro [19,20], and variables were removed sequentially if the condition index was above 30 and at least two proportions of variance, not including the intercept, was above 0.5. Interaction terms were evaluated by comparing the full model with all interaction terms included to the reduced model without any interaction terms, and backwards elimination was used on the full model to remove non-significant terms (*i.e.*, *p* > 0.05). Confounding was assessed using the all-possible subsets approach by comparing the point estimates from this model to a reduced model without the variable of interest. If the estimate for the reduced model differed by 10% from the full model, then the variable was retained in the model.

## 3. Results and Discussion

### 3.1. River Characteristics

Water quality river parameters are summarized by river and village in Table 1. While there is variability in the measurements, relative to the other rivers, the Santiago is generally characterized as fast-flowing water with high DO_2_ levels and low *E. coli* concentrations, whereas the Onzole has slow-moving water with low DO_2_ levels and high *E. coli* concentrations. The Cayapas is intermediate for all parameters except turbidity, which is lower than the Santiago. The temperatures of the Santiago and Cayapas are similar, with both running cooler than the Onzole. The Onzole has far higher turbidity levels than either of the other rivers. Villages on the same river share similar characteristics, with no statistically significant differences from one another for any of the parameters considered.

### 3.2. Impact of Distance from Shore on Water Quality

We observed significantly higher geometric mean *E. coli* concentrations within the eddy *versus* the main flow of the river (*p* = 0.0243) for the Santiago and Cayapas Rivers (Table 2), with an average within-transect paired difference of 0.12 log_10_ (*n* = 8). The Onzole was excluded from this analysis for lack of observable eddies due to low flow. Average paired *E. coli* concentrations decreased by 0.27 log_10_ (*n* = 15) between the sample closest to shore and any sample >6 m from shore.

**Figure 2:**
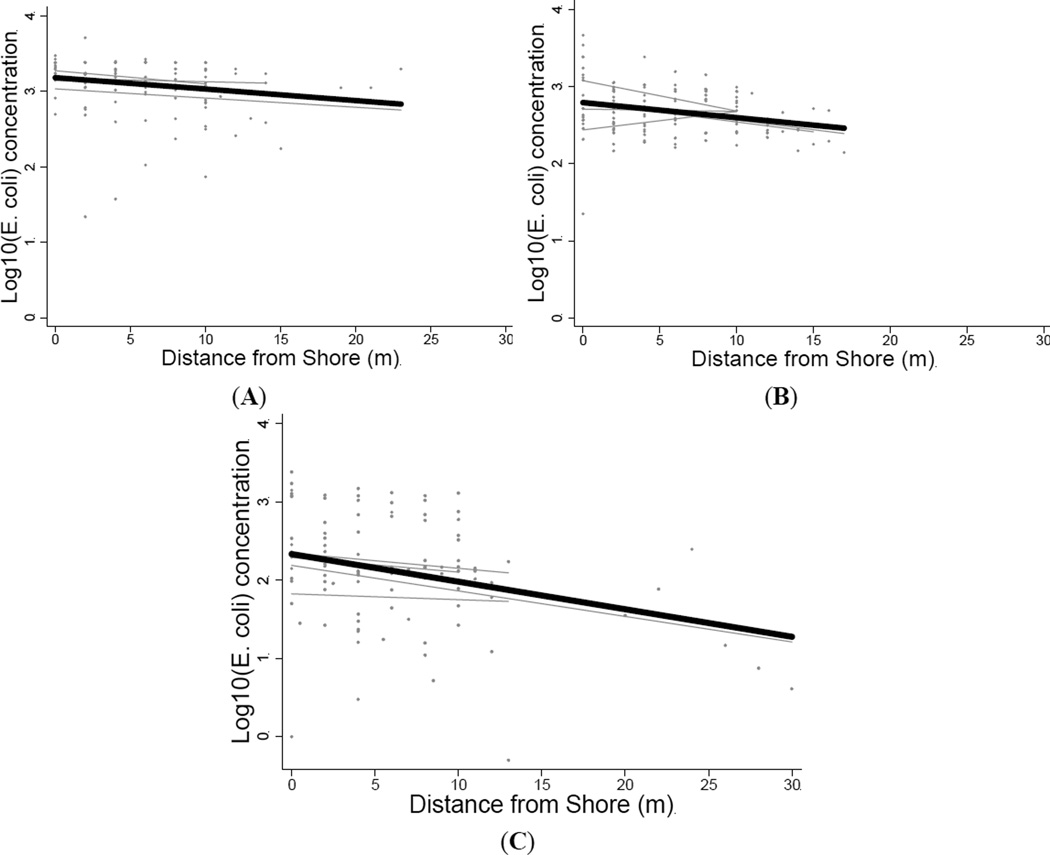
Log_10_ *E. Coli* concentration *versus* distance from shore. Best-fit lines are shown for each transect (light grey) and for each river (black) for the (**A**) Onzole; (**B**) Cayapas; and (**C**) Santiago Rivers in northern coastal Ecuador. The relationships were significantly inversely associated for all rivers (*p* < 0.05).

Log_10_ *E. coli* concentrations decreased with increasing distance from shore (m) in all three rivers (Figure 2). The Santiago River demonstrated the strongest association (β = −0.352, *p* = 0.003, *r*^2^ = 0.084), followed by the Cayapas River (β = −0.020, *p* = 0.003, *r*^2^ = 0.072), and the Onzole River (β = −0.015, *p* = 0.037, *r*^2^ = 0.037). However, all three rivers had a poor linear fit, as demonstrated by the *r*^2^ values.

The relationships between distance from shore and physiochemical measures of water quality are shown in Figure S1. DO_2_ increased with distance from shore for all three rivers, but this relationship was only statistically significant for the Cayapas and Onzole Rivers (Onzole: β = 0.035, *p* = 0.0003, *r*^2^ = 0.105; Cayapas: β = 0.027, *p* < 0.0001, *r*^2^ = 0.369; Santiago: β = 0.020, *p* = 0.168, *r*^2^ = 0.019). Turbidity decreased with distance from shore for all three rivers but this relationship was only significant for the Cayapas River (Onzole: β = −0.154, *p* = 0.908, *r*^2^ = 0.0001; Cayapas: β = −0.433, *p* = 0.007, *r*^2^ = 0.061; Santiago: β = −0.065, *p* = 0.900, *r*^2^ = 0.0002). There was a strong positive association between velocity and distance from shore for all three rivers (Onzole: β = 0.135, *p* = 0.0001, *r*^2^ = 0.411; Cayapas: β = 0.126, *p* < 0.0001, *r*^2^ = 0.596; Santiago: β = 0.174, *p* < 0.0001, *r*^2^ = 0.219).

### 3.3. Relationship between Water Quality Parameters and E. coli Concentrations

There was an inverse association between dissolved oxygen and log_10_ *E. coli* concentrations for all rivers, although the strength and significance of this relationship varied by river (Onzole: β = −0.109, *p* = 0.102, *r*^2^ = 0.023; Cayapas: β = −0.491, *r*^2^ = 0.093, *p* = 0.001; Santiago: β = −0.179, *p* = 0.028, *r*^2^ = 0.047). All three rivers showed a significant positive association between turbidity and log_10_ *E. coli* concentrations (Onzole: β = 0.001, *p* = 0.008, *r*^2^ = 0.058; Cayapas: β = 0.023, *p* < 0.0001, *r*^2^ = 0.321; Santiago: β = 0.013, *p* < 0.0001, *r*^2^ = 0.282). There was no significant trend between velocity and log_10_ *E. coli* concentration for any of the three rivers.

### 3.4. Analysis of Factors Associated with E. coli Concentrations

All variables were retained after the model selection process, except for the interaction terms. While velocity was not statistically significant, dropping it from the model did not significantly improve the AIC so it was retained in the model. The equation for the final model is given as:

(1) $${\left({\text{Log}}_{10}\text{E. coli}\right)}_{\text{ij}}=\beta_{0}+\beta_{1}{\text{Distance from shore}}_{1\text{ij}}+\beta_{2}{\text{Turbidity}}_{2\text{ij}}+\beta_{3}{\left({\text{DO}}_{2}\right)}_{3\text{ij}}+\beta_{4}{\text{Velocity}}_{4\text{ij}}+e_{\text{ij}}$$

where *i* = 1 − k; *k* = 15 for number of transects; and *j* = 1 − 6 points for each transect.

Results of the univariate analysis and correlated mixed model are shown in Table 3. In the unadjusted and adjusted estimates, distance from shore and dissolved oxygen were both negatively associated with log_10_ *E. coli* concentrations. Increased turbidity was associated with increased log_10_ *E. coli* concentrations. Velocity maintained a negative effect in the unadjusted model, but was not statistically significant when controlling for the other variables in the final adjusted model. Adjusting for turbidity, dissolved oxygen, and velocity we observed a 2% decrease in log_10_ *E. coli* concentrations with every meter from shore.

## 4. Conclusions

For three rivers that serve as surface drinking water sources for communities in northern coastal Ecuador, we found a modest reduction in *E. coli* concentrations with increasing distance from shore, for each river we examined and also when all three rivers were examined together. Log_10_ *E. coli* concentrations decreased by 2% with each meter from shore, controlling for other water quality variables. In addition, water in the main channel had *E. coli* concentrations on average 0.12 log_10_ lower than within eddies along the river shore and samples collected >6 m from shore had concentrations on average 0.27 log_10_ lower than those collected at the location closest to shore.

These findings suggest that localized microscale river dynamics, including stream velocity, eddies, and physiochemical water parameters, affect the levels of contamination encountered by people who depend on surface water. Collecting water farther from shore, in the main river channel rather than at the river shore, could offer a way for villagers to reduce the concentrations of microbes to which they are exposed through their drinking water. While the reductions are modest, and do not meet WHO health-based targets and microbiological performance specifications for household water treatment [21], in the absence of other water treatments this simple intervention could reduce the initial source water concentrations. In another study in this region, we found that baseline source water concentrations affected the effectiveness of chlorine water treatment [22]. In this region, community members could easily and safely implement this intervention because children and other community members commonly use canoes that are often available at river access points. However, it is important to note that this may not always be the case in other parts of the world.

The pattern of decreasing *E. coli* concentrations with increasing distance from shore has been observed in other studies [23,24], but this observation has mostly been limited to coastal environments. Mechanisms that could contribute to these reductions in *E. coli* with distance from shore include die-off, sedimentation, predation and more rapid transport away from the point source due to higher flow velocities in the main channel. Additional studies have shown that soil or sand at the shore can be a potential source of bacterial contamination [25–28], which could explain the spatial pattern we observed. However, our results do not depend on whether the source of contamination originates from people bathing and washing in the river or from reservoirs of microorganisms in riverbank sediments. In this study, we found increased oxygenation and decreased turbidity with distance from shore. Increased oxygenation has been shown in the literature to be associated with increased die-off, and high turbidity is also well known to be associated with high *E. coli* levels [15,28,29]. It is also possible that *E. coli* in the shore is associated with biofilms in sand particles, whereas there are less resilient, planktonic cells in the middle of the stream [30,31].

These results also suggest that contamination originating within a village is unlikely to accumulate downstream on a watershed scale, as reductions in microorganisms were observed on the scale of tens of meters. This suggests that locations where community members access the river may be serving as point sources of contamination that is limited to the village scale and is most relevant to localized transmission processes. Even greater reductions may occur over the larger scales that separate villages located kilometers away.

Our study had several limitations, suggesting lines of further research. We collected samples during only one season (the dry season), and we were unable to collect any volumetric stream flow data or fully characterize the complexities of hydrodynamic flows. Future studies could use a microbial tracer to see where the water is flowing to distinguish eddy lines more clearly. This would contribute to further water quality and hydrodynamic models for surface water. The observed reductions may be within measurement error or natural variability of the samples, so it would have been preferable to run samples in duplicate or triplicate to reduce this variability. However, given the logistical constraints of carrying out a study in a remote field location, our total sample size was limited and we chose to optimize the study design by evaluating more samples rather than running multiple IDEXX trays per sample. This allowed us to examine the relationships of interest across multiple sites. It should also be noted that false positive rates have been reported for Colilert with freshwater samples [32,33]. Additionally, other fecal indicator organisms, such as *Enterococci* and coliphage, should be tested to determine the generalizability of these results.

This study, along with others, suggests that a surface water body is heterogeneous. Predicting locations of low contamination may be beneficial in minimizing exposure to contamination [13,28,34–36].

## Supplementary Material

01

## Figures and Tables

**Table 1:** Summary of water quality river parameters by river and village.

River/Village	Geometric Mean *E. coli* Concentration (MPN/100 mL)	Median River Width (m)	Mean Temperature (°C)	Median Velocity (m/s)	Mean Dissolved Oxygen (ppm)	Median Turbidity (NTU)	Total # Samples
ONZOLE	1248 (21.8, 5172)	54 (35, 66)	26.1 (24.0, 28.1)	0.2 (0.0, 0.9)	6.96 (5.60, 7.89)	91.1 (32.2, 340)	120
Arenales	1041	42	25.7	0.5	7.44	101	48
Tangare	1408	57	26.4	0.06	6.65	87.9	72
CAYAPAS	474.4 (22.3, 4611)	88 (73, 93)	25.0 (24.6, 26.1)	0.3 (0.0, 1.3)	7.87 (7.06, 8.45)	7.03 (3.3, 55.9)	120
Telembi	561.6	81	25.2	0.3	7.93	6.2	48
Trinidad	424.0	89	25.0	0.3	7.84	7.6	72
SANTIAGO	128.0 (0.5, 2420)	144 (18, 166)	24.9 (23.4, 27.0)	0.8 (0.0, 2.1)	8.61 (5.79, 9.40)	19.4 (8.2, 106)	102
Rocafuerte	104.6	24	25.6	0.2	7.63	21.0	30
La Peña	139.3	160	24.5	1.1	9.05	15.9	72

Notes: Range of observed values for each river shown in parentheses.

**Table 2:** *E. coli* concentrations within the eddy *versus* in the main flow of the river for the Santiago & Cayapas Rivers.

Location	*n*	Geometric Mean	95% CL Mean	Coefficient of Variation	*p*-Value
Main Flow	85	197.3	(144.4, 269.4)	2.67	0.0243 *
Within Eddy	137	308.4	(242.8, 391.7)	2.53	-

Notes: Student’s *t*-test of the mean values was used to test significance;

* Indicates significant difference.

**Table 3:** Final unadjusted (univariate) and adjusted (multivariate) correlated mixed model assessing factors associated with log_10_ *E. coli* concentrations. Samples were correlated at the transect level.

Parameter	Unadjusted			Adjusted		
	Estimate	σ	*p*-Value	Estimate	Σ	*p*-Value
Intercept	-	-	-	4.91	0.531	<0.0001
Distance from Shore (m)	−0.026	0.005	<0.0001	−0.017	0.006	0.003
Turbidity (NTU)	0.004	0.0007	<0.0001	0.003	0.001	<0.0001
DO_2_ (ppm)	−0.4138	0.064	<0.0001	−0.31	0.07	<0.0001
Velocity	−0.151	0.071	0.0335	0.077	0.077	0.317
