# Supplementary material for: Spatial Variability of Escherichia coli in Rivers of Northern Coastal Ecuador
Source: Water (Basel). Author manuscript; Available in PMC 2015 May 18. (PMC4435963; doi:10.3390/w7020818)
Supplement: 01 [file NIHMS680004-supplement-01.pdf]

Supplementary Information

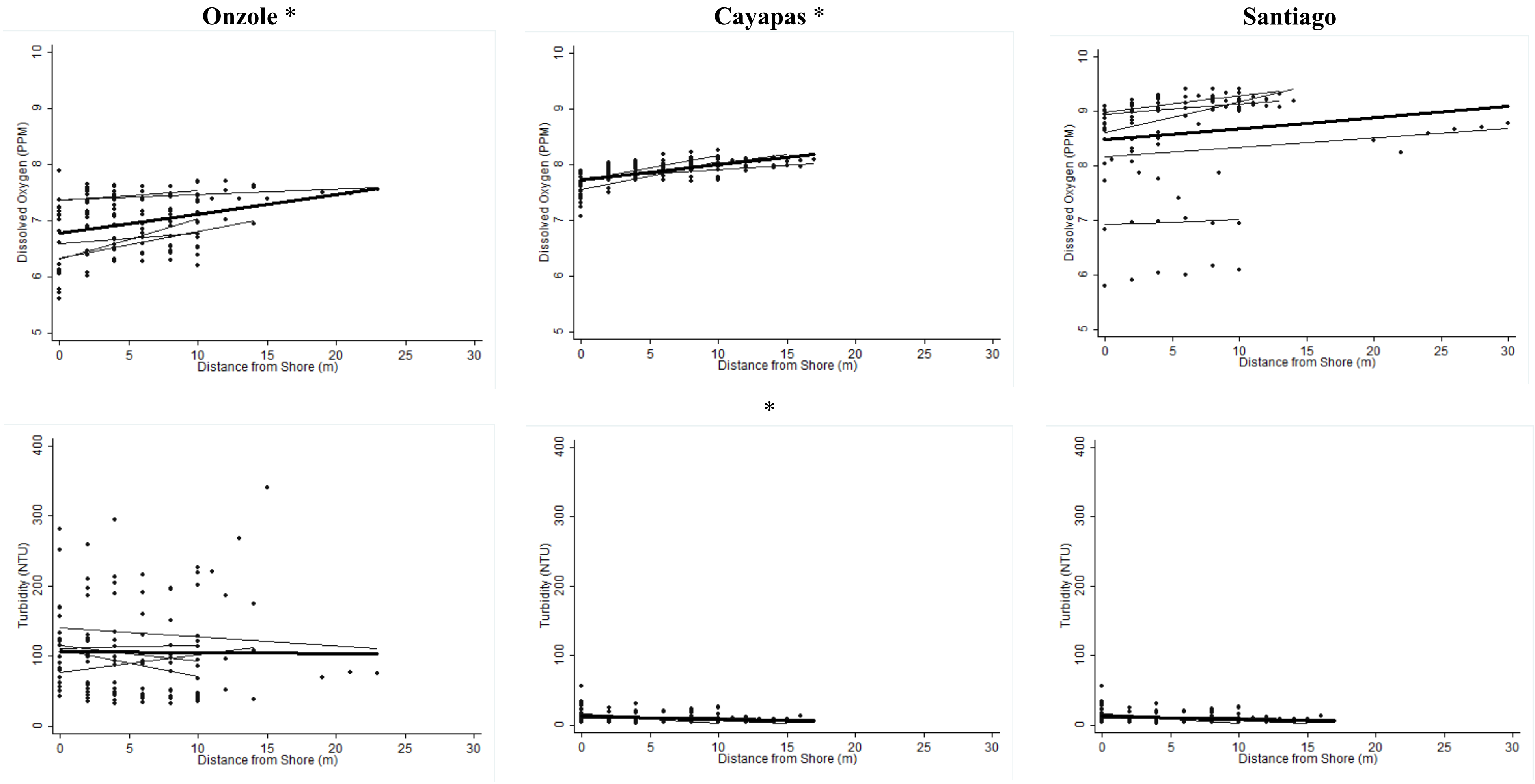

Figure S1. *Cont.*

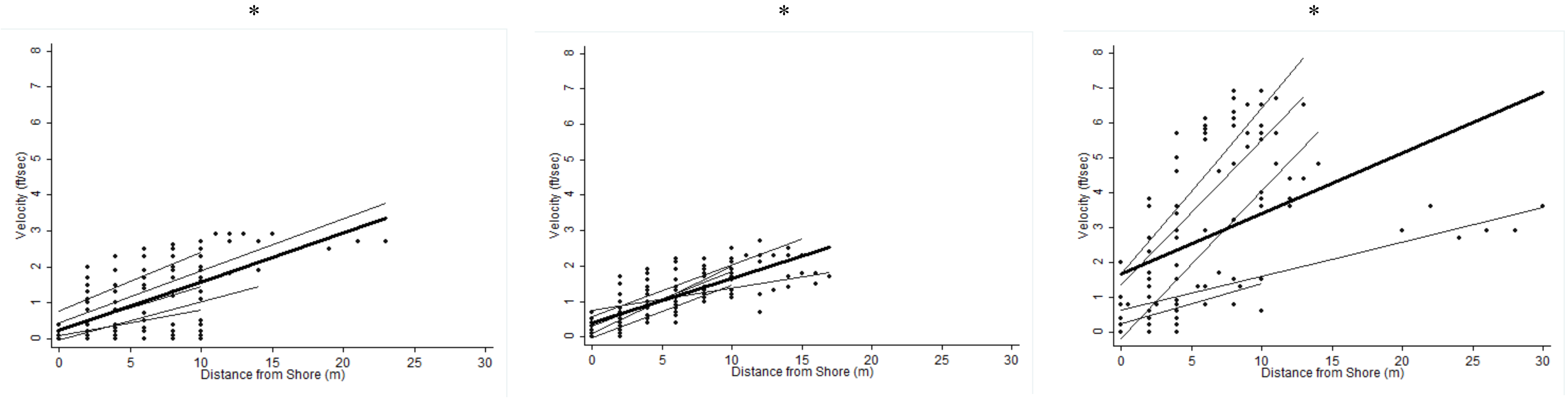

Figure S1. Overall trend of water quality variables compared to distance from shore. Best-fit lines are shown for each transect (light grey) and for each river (black). * Significant at the 95% confidence interval level.
